# Association between biomarkers and clinical characteristics in chronic subdural hematoma patients assessed with lasso regression

**DOI:** 10.1371/journal.pone.0186838

**Published:** 2017-11-06

**Authors:** Are Hugo Pripp, Milo Stanišić

**Affiliations:** 1 Oslo Centre of Biostatistics and Epidemiology, Research Support Services, Oslo University Hospital, Oslo, Norway; 2 Department of Neurosurgery, Oslo University Hospital, Oslo, Norway; Jilin University, CHINA

## Abstract

Chronic subdural hematoma (CSDH) is characterized by an “old” encapsulated collection of blood and blood breakdown products between the brain and its outermost covering (the dura). Recognized risk factors for development of CSDH are head injury, old age and using anticoagulation medication, but its underlying pathophysiological processes are still unclear. It is assumed that a complex local process of interrelated mechanisms including inflammation, neomembrane formation, angiogenesis and fibrinolysis could be related to its development and propagation. However, the association between the biomarkers of inflammation and angiogenesis, and the clinical and radiological characteristics of CSDH patients, need further investigation. The high number of biomarkers compared to the number of observations, the correlation between biomarkers, missing data and skewed distributions may limit the usefulness of classical statistical methods. We therefore explored lasso regression to assess the association between 30 biomarkers of inflammation and angiogenesis at the site of lesions, and selected clinical and radiological characteristics in a cohort of 93 patients. Lasso regression performs both variable selection and regularization to improve the predictive accuracy and interpretability of the statistical model. The results from the lasso regression showed analysis exhibited lack of robust statistical association between the biomarkers in hematoma fluid with age, gender, brain infarct, neurological deficiencies and volume of hematoma. However, there were associations between several of the biomarkers with postoperative recurrence requiring reoperation. The statistical analysis with lasso regression supported previous findings that the immunological characteristics of CSDH are local. The relationship between biomarkers, the radiological appearance of lesions and recurrence requiring reoperation have been inclusive using classical statistical methods on these data, but lasso regression revealed an association with inflammatory and angiogenic biomarkers in hematoma fluid. We thus suggest that lasso regression should be a recommended statistical method in research on biological processes in CSDH patients.

## Introduction

Chronic subdural hematoma (CSDH) is characterized by an “old” collection of blood and blood breakdown products between the brain and its outermost covering (the dura) [1, 2]. It is commonly treated by surgery, which usually results in a rapid improvement of neurological symptoms and good postoperative prognosis [2]. However, more sophisticated surgical strategies have not significantly improved outcomes over the past few decades, and there are still unacceptable rates of postoperative mortality and morbidity, most notably due to postoperative recurrence requiring reoperation (RrR). The RrR of CSDH is a major adverse postsurgical clinical outcome, which may result in repeated surgical treatments, as well as risk of further complications and mortality [3, 4].

The underlying pathophysiological process of CSDH development and progression are attributed by some studies to local immunological responses and disturbance of the coagulo-fibrinolytic system. As a result, local inflammation and angiogenesis at the site of lesions, with its corresponding pro- and anti-inflammatory cytokines,has been assessed [5–8]. Nonetheless, the association between inflammatory and angiogenic biomarkers at the site of lesions and clinical characteristics, in addition to volume of lesions, computed tomography (CT) densities and postoperative recurrence, remain unclear [9, 10]. This has limited the development of non-surgical therapeutic alternatives for CSDH patients.

We have previously used descriptive statistics and classical tests of hypotheses, and carried out work with regression analysis using a stepwise selection of predictor variables on biomarker data from CSDH [5, 6]. It is our experience that biomarker concentration data might have skewed statistical distributions, have multicollinearity issues in regression analysis due to high correlations, be associated with similar biological processes and affected by missing data. Statistical experts have reviewed the analysis of immunological data, and recommended the use of more advanced and novel statistical methods [11]. Hence, we have previously assessed the correlation between pro- and anti-inflammatory cytokines in CSDH patients using factor analysis and structural equation modeling. That study showed a strong correlation between pro- and anti-inflammatory activity in hematoma fluid samples, and a possible association with RrR [12].

Lasso regression is a statistical method that performs both variable selection and regularization. This implies mathematical procedures that tune and select the preferred level of model complexity to enhance the prediction accuracy, interpretability and generalization of the statistical model [13]. It is also used to make prediction models in a dataset with many and often inter-correlated independent variables in biological and medical research [14]. Thus, lasso regression has important statistical features to help assess the association between many biomarkers and clinical characteristics. It has been successfully applied in research on, e.g., HIV [15], neonatal sepsis [16] and bipolar disorder [17]. From a statistical point of view, it could be a very promising method to assess the association between biomarkers of inflammation in hematoma with clinical characteristics of CSDH patients in a more predictive manner, compared to previous work using factor analysis and structural equation modeling [12]. We have also applied lasso regression to develop a prognostic grading system for the prediction of CSDH RrR after initial burr-hole surgery based on demographic, radiological and surgical characteristics [18].

Biomarkers of immunological responses in hematoma fluid from a Norwegian cohort of CSDH patients were reassessed with lasso regression to explore their associations with relevant clinical characteristics and properties of the hematoma. This approach may provide additional insight into the underlying biological process of CSDH.

## Materials and methods

### Ethics statement and written informed consent

The study of human subjects was approved by the Regional Ethical Committee of the Health Region of Southeast Norway (S-06281a), while the establishment of a research bio-bank was approved by the Norwegian Directorate of Health. Written informed consent was obtained from the patients or their significant others before study inclusion. Moreover, the institutional review board approved this consent procedure.

### Patient population and management

We measured 30 selected biomarkers in hematoma fluid samples obtained during surgery of 93 adult patients with CSDH in the Department of Neurosurgery, Oslo University Hospital between January and December 2008. These patients were part of a larger cohort of 107 patients, but a biomarker analysis in the hematoma fluid was only conducted on 93 patients. Patient population, management, surgical procedure, hematoma evacuation and follow-up care are previously described [5, 6, 10].

All hematomas were classified into imaging appearance type based on density changes on CT scans, i.e., homogenous, laminar, separated, trabecular and gradation types [19]. The CSDH diagnosis by high-resolution CT scan of this cohort is thoroughly described by Stanisic and Pripp (18). Consequently, only a brief summary of this description is given as follows: The homogenous type of CSDH has homogenous density, and is categorized further into hypo, iso- or hyperdense subtypes. The laminar type has a laminar structure running along the inner membrane. The separated type has two components of different densities with a lower density component located above a higher density component, with a clear boundary lying between them. The trabecular type has inhomogeneous contents running between the inner and outer membrane on a low- to isodense background. If the boundary was indistinct, with the low and high density being mingled at the border, the CSDH was defined as the gradation type. In the gradation type, a mild head movement causes homogenization of the hematoma.

### Sample collection and measurement of biomarkers

Sample collection and the measurement of biomarkers have been previously described [5, 6], and a brief summary is given as follows: Samples from the hematoma were collected without contamination at the time of surgery. After the external membrane had been opened, a silastic catheter was inserted into the cavity of the hematoma, and approximately 10 ml of fluid was collected and aspirated into vacuum tubes that contained protamine sulfate and ethylenediamine tetraacetic acid (EDTA). All samples were immediately centrifuged at 3,000 rpm for 10 min to remove cells and debris, and the supernatants were stored in sealed polypropylene tubes at −70°C for later analysis. The concentration of 30 selected human cytokines in the samples was simultaneously determined by a multiplex antibody bead kit (Human Cytokine 30-Plex Panel for the Luminex^TM^ platform; Biosource, Camarillo, CA, USA), according to the manufacturer’s instructions. Acquired fluorescence data were analyzed by the Star Station software version 2.0 (Applied Cytometry Systems, Sheffield, UK). Furthermore, biomarker data above or below calibrated standards were discarded before further data analysis.

### Statistical analysis

The concentrations of the 30 biomarkers (after common logarithmic transformation) were described using mean and standard deviation (SD), and assessed with boxplots and histograms to examine statistical distribution. The clinical characteristics of the CSDH patients and their hematoma based on CT scan imaging were described with mean, SD and range (expressed as minimum–maximum), or the number of patients and percentage, as appropriate.

In some of the patients, there was missing data for a number of biomarkers. The lasso regression method requires complete data for all included cases. Therefore, 15 multiple imputations were iteratively conducted by chained equations using linear regression for biomarkers as implemented in the mi impute chained (regress) procedure in Stata 13.0 (StataCorp LP, College Station, TX, USA). The mean of these 15 multiple imputations was used as a single imputation for missing biomarker data. Using this approach, a complete dataset with 93 cases and 30 variables was obtained. The impact of this imputation procedure on the dataset was assessed by estimating the Cohen’s *d* effect size for each biomarker using the following formula:

$${\mathrm{Cohen}}^{'}s\;d=\frac{{\bar{x}}_{1}-{\bar{x}}_{2}}{s}$$ (1)

with the pooled SD estimated as:

$$s=\sqrt{\frac{(n_{1}-1)s_{1}^{2}+(n_{2}-1)s_{2}^{2}}{n_{1}+n_{2}-2}}$$ (2)

where n_1_ and n_2_ are the number of observations before and after imputation (the number of observations after imputation was 93 for all biomarkers), ${\bar{x}}_{1}$ and ${\bar{x}}_{2}$ are the estimated mean before and after imputation and s_1_ and s_2_ are the estimated SD before and after imputation, respectively.

Lasso regression modeling was performed with a 10-fold cross-validation, as implemented in the R package glmnet using a binomial or Gaussian response type for binary or continuous dependent variables, respectively [20]. It was done separately using the dataset with imputed values (i.e. 93 cases and 30 biomarkers) or a sub-dataset with only biomarkers with complete observed data (i.e. 93 cases and 16 biomarkers). Lasso is a penalized method for restricting the residual sum of squares (deviance) and constraining the sum of the absolute values of the regression coefficients: For a binomial or continuous dependent variable, the outcome Y is either the original dependent variable Y (cf. linear regression) or Y = log [p/(1-p)], with p as the probability of the binary event (cf. logistic regression), respectively. A full regression model for k independent variables with coefficients β is:

$$Y=\beta_{0}{+\beta }_{1}X_{1}+\beta_{2}X_{2}+\beta_{3}X_{3}+\cdots +\beta_{k}X_{k}$$ (3)

The sum of absolute values of the coefficients is then estimated with the following restriction:

$$|\beta_{0}|+|\beta_{1}|+|\beta_{2}|+\cdots +|\beta_{k}|=\sum_{i=1}^{k}|\beta_{i}|\leq \lambda$$ (4)

where *λ* is the “tuning” parameter. As λ approaches indefinite, it has no effect and the solutions are estimates for the full and unrestricted model. For smaller *λ* values, solutions are shrunken versions of the estimates, with many coefficients decreased to the null value. The selected value of λ was defined using cross-validation. The k independent variables are prior to fitting the model standardized to a mean of 0 and a standard deviation of 1. The solution of this fitted regression model is then presented with coefficients returned to the original scale, but typically with many coefficients decreased to the null value.

To investigate a robust statistical association by lasso regression, at least 50 repeated 10-fold cross-validations were performed for each model. If we found instances of cross-validation estimations with no value of λ minimizing cross-validation error, and thereby all coefficients decreased to the null value, and/or the cross-validated models had problems and warnings about convergence issues, we assumed no robust statistical association and the results from these regression analyses were not presented. However, for models that did not have such issues in repeated cross-validations, a robust statistical association was assumed. For these models, 100 repeated 10-fold cross-validations were conducted. The optimal λ (i.e. the value of λ minimizing cross-validation error) and corresponding coefficient estimates of the biomarkers from each round of cross-validations were recorded. We reported the mean, SD and range (expressed as minimum–maximum) of the coefficients, and of the optimal λ-values from these 100 rounds of cross-validation estimations. Median values were also estimated, but were similar to the mean values. Therefore, the mean was reported. The models had both predictive ability and a limited number of selected coefficients. Because the biomarkers were selected into the regression model based on minimizing cross-validation error, p-values for each of the selected biomarkers are not relevant and hence not estimated. We assessed the predictive performance of the selected models by estimating the area under the receiver operation curve (AUC) for binary responses using the R package pROC [21], and reported the mean, SD and range of the estimated AUC values from the 100 rounds of cross-validation estimations. For a more comprehensive and technical discussion of lasso regression methodology, see, e.g., [22].

We performed multiple imputations of missing data with Stata 13.0 (StataCorp LP, College Station, TX), and descriptive statistics and lasso regression with R version 3.2.0 (R Foundation for Statistical Computing, Vienna, Austria).

## Results

Table 1 shows the clinical characteristics of the 93 CSDH patients, with classification based on CT scan imaging appearance and pre- and postoperative volume of hematomas. This cohort of 93 CSDH patients had a mean age 72.2 (SD 12.3) within the range from a minimum of 34 to a maximum of 90 years, with a dominant proportion of males (64.5%) and a high prevalence of neurological symptoms and signs as expressed by motor- and speech deficiency, dementia, preoperative Glasgow Coma Scale (GCS) and prevalence of brain infarct. The homogenous type of CSDH was most frequent (55.9%), followed by trabecular (19.4%), separated (8%), gradation (8%) and laminar (7%). The volume was reduced by approximately 50 mL as a result of surgical treatment.

**Table 1:** Study population characteristics of CSDH patients (n = 93).

Clinical characteristics		
Mean age (SD) [range]	Years	72.2 (12.3) [34–90]
Gender	Males	60 (64.5%)
	Females	33 (35.5%)
Preoperative GCS score	3–14	58 (62.4%)
	15	35 (37.6%)
RrR	Yes	16 (17.2%)
	No	77 (82.8%)
Brain infarct	Yes	18 (19.4%)
	No	75 (80.6%)
Motor deficiency	Yes	58 (62.4%)
	No	35 (37.6%)
Speech deficiency	Yes	31 (33.3%)
	No	62 (66.7%)
Dementia	Yes	7 (7.5%)
	No	86 (92.5%)
**CSDH based on CT scan imaging**		
Homogenous type	Yes	52 (55.9%)
	No	41 (44.1%)
Hypodense homogenous subtype	Yes	27 (29.0%)
	No	66 (71.0%)
Isodense homogenous subtype	Yes	13 (14.0%)
	No	80 (86.0%)
Hyperdense homogenous subtype	Yes	12 (12.9%)
	No	81 (87.1%)
Laminar type	Yes	7 (7.5%)
	No	86 (92.5%)
Separated type	Yes	8 (8.6%)
	No	85 (91.4%)
Trabecular type	Yes	18 (19.4%)
	No	75 (80.6%)
Gradation type	Yes	8 (8.6%)
	No	85 (91.4%)
CSDH densities with high risk for RrR	Yes ^a^	40 (43.0%)
	No ^b^	53 (57.0%)
Mean preoperative volume (SD)[range]	mL	154.3 (73.0)[24.3–380.1]
Mean postoperative volume (SD)[range]	mL	104.6 (57.3)[18.7–306.6]

CSDH, chronic subdural hematoma; SD, standard deviation; GCS, Glascow Coma Scale; RrR, recurrence requiring reoperation; CT, computed tomography.

^a^ Isodense- or hyperdense homogenous subtypes and laminar- or separated types.

^b^ Hypodense homogenous subtypes and trabecular or gradation type.

Table 2 shows the concentrations of the 30 biomarkers in log pg/mL in hematoma fluid samples, both before and after the imputation of missing data. Sixteen of the 30 selected biomarkers (53.3%) had complete observed data for all of the included 93 patients. TNF-α had the most missing data, with missing values for 28 patients (30.1%). Nineteen patients (20.4%) had complete observed data for all 30 biomarkers, 61 patients (65.6%) had missing values for one to three biomarkers and two patients (2.1%) had missing data for seven biomarkers, which was the highest number of missing data among the included patients. Overall, the mean concentrations were somewhat lower in the dataset after imputation, except for IL-6, TNF-α and CXCL10, with a higher mean concentration after imputation. Based on the estimated Cohen’s *d*, IL-6, EGF, IL-1β and IL5 were mostly affected by imputation. The SD was affected to a small degree by the imputation of missing data. IL-6, CXCL8, CCL2 and HGF had mean concentrations above 3.0 log pg/mL, while on the other hand IL-2, IL-4, TNF-α and EGF had mean concentrations below 1.0 log pg/mL.

**Table 2:** Concentration of biomarkers (log pg/mL) in CSDH fluid samples before and after the imputation of missing data.

Biomarkers ^a^		Before imputation	After imputation	Effect size imputation
	N	Mean (SD)	Mean (SD)	Cohen’s *d*
IL-1β	70	1.24 (0.74)	1.09 (0.76)	0.20
**IL-1RA**	93	2.64 (0.44)	2.64 (0.44)	
IL-2	76	0.66 (0.66)	0.57 (0.65)	0.14
**IL-2R**	93	2.76 (0.35)	2.76 (0.35)	
**IL-4**	93	0.98 (0.41)	0.98 (0.41)	
IL-5	80	1.37 (0.53)	1.27 (0.57)	0.18
IL-6	69	3.72 (0.83)	3.92 (0.85)	-0.24
**IL-7**	93	1.72 (0.17)	1.72 (0.17)	
**CXCL8 (IL-8)**	93	3.43 (0.81)	3.43 (0.81)	
**IL-10**	93	1.33 (0.37)	1.33 (0.37)	
**IL-12**	93	1.94 (0.32)	1.94 (0.32)	
IL-13	83	1.33 (0.48)	1.27 (0.50)	0.12
**IL-15**	93	1.86 (0.37)	1.86 (0.37)	
IL-17	72	1.34 (0.58)	1.34 (0.54)	0
TNF-α	65	0.48 (0.44)	0.51 (0.43)	-0.07
IFN-α	91	1.59 (0.31)	1.58 (0.31)	0.03
**IFN-γ**	93	1.25 (0.39)	1.25 (0.39)	
GM-CSF	84	1.38 (0.40)	1.35 (0.40)	0.07
**CCL3 (MIP-1α)**	93	1.63 (0.25)	1.63 (0.25)	
**CCL4 (MIP-1β)**	93	1.56 (0.44)	1.56 (0.44)	
CXCL10 (IP-10)	86	2.50 (0.51)	2.51 (0.50)	-0.02
CXCL9 (MIG)	89	1.85 (0.56)	1.84 (0.55)	0.02
Eotaxin	86	1.28 (0.47)	1.26 (0.49)	0.04
**CCL5 (RANTES)**	93	2.08 (0.55)	2.08 (0.55)	
CCL2 (MCP-1)	90	3.55 (0.54)	3.55 (0.53)	
**VEGF**	93	2.37 (0.49)	2.37 (0.49)	
EGF	77	0.59 (0.62)	0.45 (0.66)	0.22
**G-CSF**	93	2.11 (0.55)	2.11 (0.55)	
**FGF-basic**	93	1.37 (0.36)	1.37 (0.36)	
**HGF**	93	3.59 (0.31)	3.59 (0.31)	

CSDH, chronic subdural hematoma; N, number of patients with observed concentration of the biomarker; SD, standard deviation.

^a^ The 16 biomarkers in bold letters had complete observed data for all included 93 patients (i.e., complete data subsample).

Table 3 presents coefficients from lasso regression modeling on the relationship between biomarkers and characteristics of the study population described in Table 1. Based on our criteria for a robust model using multiple cross-validations, no robust statistical association was found with the concentration of biomarkers and age, gender, preoperative GCS score, brain infarct, motor- or speech deficiency, dementia, pre- or postoperative volume of hematoma, and most types of CSDH based on CT scan imaging. Thus, all coefficients were set to zero in some instances of the lasso regression model after cross-validation and/or there were problems with convergence of the statistical models. Models with these statistical issues are therefore not reported in Table 3, and were deemed to express a lack of statistical association between the concentration of biomarkers and clinical or CT scan imaging characteristics. However, lasso regression models exhibited a robust statistical association between the biomarkers and RrR, as well as the combination of CSDH densities previously defined as high risk for RrR [10]. A robust statistical association was also found for hypodense homogenous subtype (only complete data subsample), hyperdense homogeneous subtype (only dataset with all 30 biomarkers) and the trabecular type (only complete data subsample). The lasso regression models had a reasonable model fit with the most AUC above 0.80.

**Table 3:** Mean, (SD) and range expressed as [minimum–maximum] of lasso coefficients of biomarkers on characteristics of CSDH patients described in Table 1 from 100 rounds of cross-validation estimations.

Characteristic	RrR		CSDH densities with high risk for RrR		Hypodense homogenous subtype	Hyperdense homogenous subtype	Trabecular type
Biomarkers^a^	All	Complete data subsample	All	Complete data subsample	Complete data subsample	All	Complete data subsample
IL-1β						-0.05 (0.24) [-1.47–0]	
**IL-1RA**	0 (0.01) [-0.12–0]				0.31 (0.09) [0–0.56]	-0.04 (0.19) [-1.33–0]	
IL-2	-0.04 (0.13) [-0.79–0]					0 (0.03) [0–0.19]	
**IL-2R**			-0.41 (0.07) [-0.47–0]	-0.84 (0.16) [-1.01–0]	0.24 (0.05) [0–0.33]		
**IL-4**					-0.03 (0.06) [-0.33–0]		0.59 (0.19) [0–0.85]
IL-5	-0.34 (0.07) [-0.72 –-0.26]		-0.29 (0.05) [-0.36–0]				
IL-6			0 (0.01) [0–0.03]			0 (0.01) [0–0.08]	
**IL-7**			0.85 (0.14) [0–1.00]	1.19 (0.23) [0–1.45]	-3.32 (0.88) [-5.28–0]	0.07 (0.37) [0–2.46]	0.26 (0.28) [0–1.64]
**CXCL8 (IL-8)**	-0.58 (0.13) [-1.25 –-0.46]	-0.62 (0.05) [-0.62 –-0.36]	-0.70 (0.13) [-0.87–0]	-0.59 (0.12) [-0.77–0]	0.02 (0.03) [0–0.12]	-0.67 (0.19) [-1.46 –-0.44]	0.62 (0.15) [0–0.85]
**IL-10**		-0.50 (0.05) [-0.62 –-0.36]					0.23 (0.14) [0–0.68]
**IL-12**					0.97 (0.27) [0–1.51]	0 (0.03) [0–0.29]	-0.02 (0.07) [-0.59–0]
IL-13	-0.09 (0.06) [-0.24–0]						
**IL-15**			-0.01 (0.02) [-0.07–0]				
IL-17						-0 (0.01) [-0.06–0]	
TNF-α						0.07 (0.34) [0–2.24]	
IFN-α	0 (0.03) [-0.23–0]						
**IFN-γ**	-0.86 (0.11) [-0.93 –-0.27]	-1.01 (0.04) [-1.06 –-0.83]		-0.04 (0.03) [-0.15–0]			
GM-CSF	-0.03 (0.09) [-0.61–0]						
**CCL3 (MIP-1α)**							
**CCL4 (MIP-1β)**					0.42 (0.16) [0–0.83]		-0.65 (0.35) [-1.30–0]
CXCL10 (IP-10)	-0.17 (0.05) [-0.35 –-0.11]						
CXCL9 (MIG)							
Eotaxin							
**CCL5 (RANTES)**	0.53 (0.04) [0.43–0.57]	0.55 (0.02) [0.45–0.56]	0.01 (0.01) [0–0.04]	0.24 (0.04) [0–0.27]	-0.93 (0.21) [-1.28–0]	0.47 (0.03) [0.36–0.52]	0.33 (0.18) [0–0.74]
CCL2 (MCP-1)			0.54 (0.09) [0–0.65]			0.14 (0.14) [0–0.51]	
**VEGF**	0.07 (0.18) [0–0.76]	0.07 (0.07) [0–0.25]			0.00 (0.01) [0–0.07]	0.01 (0.03) [0–0.17]	
EGF	0.02 (0.06) [0–0.43]		0.19 (0.03) [0–0.23]			0.05 (0.10) [0–0.59]	
**G-CSF**	0.01 (0.06) [0–0.50]		0.23 (0.07) [0–0.34]	0.30 (0.09) [0–0.51]			0 (0.02) [-0.15–0]
**FGF-basic**						0.16 (0.35) [0–1.86]	
**HGF**			0.10 (0.07) [0–0.23]	0.21 (0.08)	-0.53 (0.13) [-0.81–0]	0.46 (0.29) [0–1.34]	
**Model fit**							
Optimal λ after CV	0.045 (0.008) [0.016–0.060]	0.04 (0.004) [0.031–0.054]	0.047 (0.014) [0.034–0.137]	0.045 (0.017) [0. 028–0.137]	0.038 (0.016) [0.020–0.120]	0.044 (0.011) [0.011–0.065]	0.032 (0.014) [0.015–0.098]
AUC (95% CI)	0.87 (0.01) [0.85–0.91]	0.85 (0.002) [0.85–0.85]	0.80 (0.04) [0.5–0.81]	0.77 (0.05) [0.5–0.78]	0.82 (0.06) [0.5–0.84]	0.83 (0.04) [0.78–0.96]	0.75 (0.03) [0.5–0.79]

SD, standard deviation; CSDH, chronic subdural hematoma; RrR, Recurrence requiring reoperation; CV, Cross-validation; AUC, Area under the receiver operation curve.

^a^ The 16 biomarkers in bold letters had complete observed data for all included 93 subjects (i.e. complete data subsample).

None of the presented lasso regression models included CCL3, CXCL9 and Eotaxin, and the mean coefficient from rounds of cross-validations were close to zero for IL-1β, IL-2, IL-6, IL-15, IL-17, IFN-α and GM-CSF. This may indicate that these biomarkers had a low statistical association with RrR and CSDH densities with a high risk of RrR. The fitted regression model using data from all 30 biomarkers on RrR had negative mean coefficients (i.e. a lower probability of RrR with increased concentrations) for IL-5, CXCL8, IL-13, IFN-γ and CXCL10, and positive mean coefficients (i.e. a higher probability of RrR with increased concentrations) for CCL5 and VEGF. Statistical analysis using the complete data subsample of 16 biomarkers showed a similar trend, but IL-10 was then included with a negative coefficient. For CSDH densities with high risk for RrR, a somewhat similar pattern was observed, but in addition there was a negative coefficient for IL-2R, a positive coefficient for IL-7, CCL5, CCL2, EGF, G-CSF and HGF and no statistical association with IL-13 and a very low association for IFN-γ. Multiple cross-validation of the lasso regression model for trabecular type showed that only the complete data subsample of 16 biomarkers yielded robust statistical models. The regression model for trabecular type (complete data subsample) revealed positive associations for IL-4, IL-7, CXCL-8, IL-10 and CCL5 and negative associations for CCL4, and to a certain degree for IL-12. Coefficients from the models for hypodense (complete data subsample) and hyperdense homogenous subtype are also shown, as these did not have problems during multiple cross-validations. Still, regarding the lack of a robust statistical association for most of the CSDH based on CT scan imaging (Table 1), these results should be interpreted with caution.

We conducted multiple rounds of cross-validation, and recorded the optimal λ-value and corresponding coefficient estimates, to help assess the robustness of the selected biomarkers. IL-5, CXCL-8, IL-10 (only complete data subset), IFN- γ, CXCL-10 and CCL5 were selected in all rounds of cross-validation for fitting a regression model on RrR. Their SDs from these rounds of cross-validation were low compared to their mean values. This indicates a robustness of these selected biomarkers. The robustness in selecting biomarkers seems not be as strong as for the other reported regression models. However, both CXCL8 and CCL5 were selected in all rounds of cross-validation for fitting a model on the hyperdense homogenous subtype. IL-2R, IL-5, IL-7, CXCL8, CCL5 (only for complete data subset), CCL2, EGF and G-CSF seemed reasonably robust for the regression models on CSDH densities with a high risk of RrR.

Comparing models based on all 30 biomarkers, or on the complete data subsample of 16 biomarkers, CXCL8, INF-γ and CCL5 were consistently selected in models on RrR. A similar degree of consistency between all, and the complete data subsample of 16 biomarkers, was found for IL-2R, IL-7, CXCL8 and G-CSF in lasso regression models on CSDH densities with a high risk of RrR. By contrast, IL-10 was robustly selected in the model on RrR using the complete data subsample, but not using all biomarkers.

## Discussion

We have previously found that the correlation of inflammatory processes between blood and hematoma fluid samples were low, and that the immunological responses occur both locally at the site of CSDH and systemically in patients with CSDH [5, 12]. However, our previous statistical studies on the association between the concentration of specific biomarkers and patient clinical characteristics, and types of CSDH based on CT scan imaging, have been inconclusive. Many variables compared to the number of observations, missing data, collinearity (i.e. a high correlation between biomarkers) and skewed distributions have been statistical obstacles, using classical linear or logistic regression analysis to assess these associations. On the other hand, we have shown an association between clinical data on hematoma volume, hematoma characteristics based on CT scan imaging appearance and RrR [18]. Compared to previous work with factor analysis and structural equation modeling [12], statistical analysis with lasso regression is targeted more on prediction. It also has the ability to exclude biomarkers from the statistical model with a low association with assessed outcome. That feature was very useful when we analyzed all 30 biomarkers in this study.

### Lasso regression on clinical presentation of CSDH patients

Lasso regression showed a lack of association between these 30 biomarkers in hematoma fluid, with many relevant clinical characteristics of CSDH patients such as age, gender, neurological deficiencies and hematoma volume. For this reason, immunological responses expressed by the 30 biomarkers seemed to not be associated with clinical characteristics of the CSDH patients and the volume of hematoma. Moreover, it corresponds with previous studies on this cohort that also demonstrated a low correlation of the immunological response between blood and the hematoma fluid, and that the immunological response occurred locally in the hematoma [5, 6, 12].

### Relationship with postoperative RrR

RrR remains a crucial problem associated with current surgical treatment, and occurs in 2.5% to 33% of cases [4, 23–25]. We found a robust statistical association with RrR using lasso regression. These findings indicate that even if the 30 biomarkers were not deemed to be statistically associated with neurological deficiencies or volume of hematoma, they could be associated with RrR. In a recent study with this patient cohort, we have shown that CSDH densities and pre- and postoperative cavity volume are strong predictors of RrR [18]. Factor analysis and structural equation modeling have revealed an association between inflammatory and angiogenic activities in hematoma fluid and RrR [12].

The pro-inflammatory cytokine CXCL8 in Table 3 had a large coefficient and thus, statistically speaking, a likely association with RrR. Yet, it had a negative coefficient, thereby indicating that an increased CXCL8 reduced the risk of RrR. This somewhat contradicts findings by other studies on CSDH and RrR. Increased IL-6 and CXCL8 concentrations in CSDH fluid have been attributed to CSDH pathogenesis and inflammatory reaction of the dural border layer cells [26], and a correlation between inflammatory activity and CSDH recurrence has been identified [27]. Another study has found that patients with high concentrations of anti-inflammatory cytokine IL-10 also had high values for IL-6 and CXCL8, in addition to a tendency to be associated with a separated or layer type of hematoma [28]. It has been proposed that CXCL8 inducing a neutrophil respiratory burst is the crucial impact when subdural effusion develops into CSDH, and that an increased concentration of CXCL8 may increase the risk of RrR [29]. We found though that the concentration of CXCL8 was high compared to the other assessed biomarkers (Table 2). It could be that CXCL8 has an important role in the development of CSDH, but its relation to RrR needs further investigation. The incidence of RrR was 42.8%, 25%, 19.2% and 5.5% for laminar, separated, homogenous and trabecular type, respectively. Consequently, the inverse relationship between CXCL8 and RrR could be due to its association with types of hematoma densities with a lower risk of RrR in this cohort. A larger and new dataset is needed to validate these findings, and to provide a better understanding.

Our results indicated that IL-5, IL-13, IFN-γ and CXCL10 were negatively associated with RrR (Table 3). Both IL-5 and IL-13 have assumed to be related to pathological responses [30], and have been linked to the development of lung diseases [31]. However, as for CXCL8, they have a somewhat unexpected negative association with the development of RrR, while the association with IL-13 was also weak in the statistical model. These results should therefore be interpreted with some caution, and validations with new and larger datasets are warranted. Even so, it may reflect that the immunological responses in hematoma might follow a different pattern compared to typical systemic diseases. We have not identified studies on the specific role of IFN-γ in CSDH. However, in early human fracture hematoma, this pro-inflammatory cytokine together with CXCL8 had elevated concentrations [32]. As discussed above for CXCL8, it could therefore be assumed that INF-γ as a pro-inflammatory biomarker would be positively correlated with RrR. The lasso regression model somewhat contradicts this with negative coefficients for both biomarkers. Again, a new and larger dataset is needed to validate these results and to give a better understand of its role in CSDH. Yet, the statistical analysis in this cohort indicates that INF-γ may have a role in pathogenesis. CCL5, also known as RANTES, was positively associated with RrR. In a study on traumatic brain injury patients, a significant correlation of admission CCL5 levels in plasma and early mortality was found [33]. An elevation in human fracture hematomas and the surrounding bone marrow obtained from immunological restricted patients was also found compared to controls [34]. Thus, it may be speculated that elevated levels of CCL5 are associated with CSDH patients with immunological impairment, which makes them suffer a delayed and insufficient healing, and thus a higher risk of RrR.

### Biomarkers and hematoma densities

Multiple rounds with cross-validation showed a lack of robust statistical association for several of the main types based on CT imaging. A robust statistical relationship for the hypodense homogenous subtype and the trabecular type was only found using the complete data subsample of 16 biomarkers. A correlation of the beta-trace protein and inflammatory cytokines IL-6 and CXCL8 with magnetic resonance imaging in CSDH has been reported [35]. However, another study found that the levels of IL-6, TNF- α, and IL-10 were extremely high, but had no significant differences in relation to the CT features [7]. Our results on the association between types based on CT imaging and biomarkers should be interpreted with caution. Samples may also have been drawn from different locations of the more heterogeneous subdural fluid collections, which may have differences in inflammatory markers.

### Lasso regression as statistical method in research on CSDH

Except for our recent study on prognostic factor using this patient cohort [18], lasso regression has not to the best of our knowledge been applied in studies on CSDH. Still, as previously described, an increasing application in the assessment of biomarkers for other medical conditions has also been discovered. We found lasso regression to be promising compared to more classical approaches, such as, e.g., stepwise regression, which in an initial analysis did not robustly estimate a logistic regression model for RrR using all 30 biomarkers (results not shown). It has been argued in statistical literature that even if stepwise variable selection is a very popular technique used for many years, had this procedure just been proposed as a statistical method, it would have most likely been rejected because it violates principles of statistical estimation and hypothesis testing [36]. For instance, the drawbacks of stepwise regression are shown to include parameter estimations with standard errors of regression estimates that are biased low and confidence intervals for effects and predicted values that are falsely narrow, in addition to inconsistencies among model selection algorithms and problems of multiple hypothesis testing [37].

For example, missing data can be a problem in the assessment of biomarkers due to technical limitations or problems during laboratory analysis. The lasso regression model requires complete data to include a case. Thus, a patient with missing data for one or more biomarkers will be excluded. In our dataset, there were only 19 patients with complete data for all 30 biomarkers, which only represented a very small subset of the cohort. A subsample of 19 patients with complete data was deemed too small, and with too few subjects having specific events, such as, e.g., RrR. We therefore used a subsample of 16 biomarkers with complete observed data for all 93 patients. The results from lasso regression with only these 16 biomarkers were comparable to an analysis based on imputed data, so the results from this subsample of biomarkers would not be affected by missing data. We hence have the most statistical confidence in biomarkers that were selected in the models using all 30 biomarkers and the complete data subsample of 16 biomarkers. There has been some development in methodology for missing data and lasso regression. A Method using a stochastic EM algorithm for generalized linear models with penalized maximum likelihood parameter estimation in the presence of missing data has been proposed [38]. There is a quite amount of work in a general resampling approach for variable selection in the presence of missing data [39], as well as high-dimensional variable selection in regression and classification with missing data [40]. However, these methods seem somewhat limited in their implementation, and are not yet integrated into the glmnet R package. Instead, we chose a somewhat pragmatic approach, and used a single imputation strategy based on data from multiple imputations. A single imputation approach made it possible to use all methods integrated into the R package glmnet for lasso regression. Even so, the influence of missing data and the use of imputation techniques in lasso regression need further investigation and validation.

We found that conducting multiple rounds of cross-validation, selecting the optimal λ-value and recording the corresponding lasso coefficients was a useful approach to help assess the robustness of the models. Based on estimated coefficients and predictive ability, the lasso regression model on RrR, with both using all 30 biomarkers or the complete data subset of 16 biomarkers, seemed most robust and consistent among the investigated regression analysis. The variation in selected optimal λ-values and corresponding lasso coefficient estimates from each round of cross-validation might reflect the relatively low sample size compared to number of variables in the models. Using a leave-one-out cross-validation approach instead of the default 10-fold cross-validation would give identical λ-values and corresponding lasso coefficients in each round. However, 10-fold cross-validation is the default method in the R package glmnet [20]. Again, a new and larger sample with biomarkers from CSDH patients is needed to further assess robustness and consistency. Even so, comparing results from multiple rounds of cross-validation provides an indication of robustness and consistency.

### Proposed mechanism of the immunological processes and clinical characteristics of CSDH

We propose that immunological processes, as expressed by the investigated biomarkers, are associated with RrR. The internal relationship between CXCL8, IFN-γ and CCL5 in particular may express an immunological impairment for CSDH patients. This immunological impairment could result in a delayed and insufficient healing, and put them at higher risk of RrR. Furthermore, we propose that other clinical characteristics of CSDH, such as preoperative GCS score and neurological deficiencies, are not directly related to the specific immunological process at the site of hematoma. Volume, the localization of lesions and other comorbidities could be of more importance for these clinical characteristics. We suggest that lasso regression might be a recommended statistical method in ongoing research on biological processes in CSDH patients. Nevertheless, our statistical modeling needs further validation and preferably on a new dataset with CSDH patients. At present, we do not have access to any new data on biomarkers in CSDH patients, but we are planning to pursue further research in this field.

## Supporting information

S1 Text Concerning sharing of research data. (DOCX)
