# Supplementary material for: Association between biomarkers and clinical characteristics in chronic subdural hematoma patients assessed with lasso regression
Source: PLoS One. 2017 Nov 6;12(11):e0186838. doi: 10.1371/journal.pone.0186838 (PMC5673201; doi:10.1371/journal.pone.0186838)
Supplement: S1 Text — (DOCX) [file pone.0186838.s001.docx]

**Concerning sharing of research data**

This document is a statement on the processing of personal data in a specific research project according to Norwegian law, and in accordance with Directive 95/46/EC of the European Parliament and of the Council of 24 October 1995 on the protection of individuals with regard to the processing of personal data and on the free movement of such data.

In accordance with The Personal Data Regulations § 7-12, The Data Protection Officer has the authority to recommend the data processing in research projects, as notified by the Project Manager. The recommendation is given with several conditions, whereof the most vital is hereby translated. Failure to comply with these conditions will define the treatment of data as illegal:

1. Personal data and research data is defined as any information that can be traced back to individuals. This includes coded, deidentified data. As far as there exists any possibility to identify the individual in the data, the data is regulated as personal information. It's not relevant in this context that those who have access to information are not able to identify individuals directly. What matters is that such a possibility exists at all.

2. Data can only be processed within the purpose as specified in the notification to The Data Protection Officer, and as stated in the informed consent. Processing of data for new or changed purposes, requires a renewed informed consent and a notification to the Data Protection Officer.

3. The informed consent is given to Oslo University Hospital, who thus has the responsibility as Data Controller. The hospital is obliged to ensure that data is handled within the stated purpose, and on equipment that is under the hospital's authority.

4. Consequently, handing over research data to journals in order to submit a publication, is not covered by this recommendation. Such action would be contrary to Norwegian law. If required, provisions can be made for the inspection of the data as long as the data is under the hospital's control, hence the Data Controller-responsibility.

Questions may be directed to The Data Protection Officer; [personvern@ous-hf.no](mailto:personvern@ous-hf.no) .

Reference: The Norwegian Data Protection Authority; <https://www.datatilsynet.no/English/> .

Office of the Privacy and Data Protection Officer

Executive Staff

Oslo University Hospital HF

Email: [personvern@ous-hf.no](mailto:personvern@ous-hf.no)

www.oslo-universitetssykehus.no/personvern
